# Histamine dihydrochloride and low-dose interleukin-2 in an emerging landscape of relapse prevention in acute myeloid leukemia

**DOI:** 10.1177/20406207251351086

**Published:** 2025-06-28

**Authors:** Malin S. Nilsson, Anna Martner, Lovisa Wennström, Markus Hansson, Fredrik B. Thorén, Kristoffer Hellstrand

**Affiliations:** TIMM Laboratory, Sahlgrenska Center for Cancer Research, University of Gothenburg, Gothenburg, Sweden; Department of Microbiology and Immunology, Institute of Biomedicine, Sahlgrenska Academy, University of Gothenburg, Gothenburg, Sweden; TIMM Laboratory, Sahlgrenska Center for Cancer Research, University of Gothenburg, Gothenburg, Sweden; Department of Microbiology and Immunology, Institute of Biomedicine, Sahlgrenska Academy, University of Gothenburg, Gothenburg, Sweden; Department of Hematology, Sahlgrenska University Hospital, Gothenburg, Sweden; Department of Hematology, Sahlgrenska University Hospital, Gothenburg, Sweden; TIMM Laboratory, Sahlgrenska Center for Cancer Research, University of Gothenburg, Gothenburg, Sweden; Department of Medical Biochemistry and Cell Biology, Institute of Biomedicine, Sahlgrenska Academy, University of Gothenburg, Gothenburg, Sweden; TIMM Laboratory, Sahlgrenska Center for Cancer Research, University of Gothenburg, Box 425, 40530 Gothenburg, Sweden; Department of Infectious Diseases, Institute of Biomedicine, Sahlgrenska Academy, University of Gothenburg, Gothenburg, Sweden

**Keywords:** acute myeloid leukemia, histamine dihydrochloride, immunotherapy, relapse prevention, remission maintenance

## Abstract

Effective strategies to maintain complete remission in adults with acute myeloid leukemia (AML) are critically needed. Early clinical trials aimed at preventing relapse in the postconsolidation phase explored prolonged chemotherapy, single-agent immunotherapy, and hybrid chemo-immunotherapy, but none of these approaches produced practice-changing results. More recent trials have identified efficacious remission maintenance strategies, including (1) midostaurin or quizartinib for patients with *FLT3*-mutated AML, (2) oral azacitidine for older AML patients, and (3) immunotherapy with histamine dihydrochloride and low-dose interleukin-2 (HDC/IL-2) for younger patients. In this review, we examine key phase III trial and follow-up study results for approved remission maintenance therapies, with a particular focus on HDC/IL-2. We discuss clinical efficacy in relation to patient age and anti-leukemic immunity as well as leukemic cell chemosensitivity, chromosomal integrity, and mutational profiles. Finally, we propose a role for HDC/IL-2 within an evolving landscape of strategies to achieve durable remission in a broader population of AML patients.

## Background

At diagnosis, almost all patients with acute myeloid leukemia (AML) promptly receive chemotherapy, albeit of variable intensity. For fit patients, initial treatment (the induction phase) aims at achieving complete remission (CR), defined as disappearance of morphologically detectable leukemic cells along with the return of nonmalignant hematopoiesis. When in CR, patients receive consolidation chemotherapy, mostly in 2–4 repeated courses over approximately 3–6 months, to eradicate remaining leukemic cells and reduce the risk of relapse. For adult AML patients eligible for intensive treatment, the mainstay of induction and consolidation therapy is anthracyclines and/or cytarabine.^1^ After the completion of chemotherapy, patients with high-risk AML defined by cytogenetics or mutations in leukemic cells may be candidates for allogeneic stem cell transplantation (allo-SCT).^2^

The past decades have seen advances in AML therapy such as curative therapy in promyelocytic leukemia, targeted therapy for molecular subgroups of AML, use of alternative upfront chemotherapy for older patients (e.g., hypomethylating agents in combination with venetoclax), molecular monitoring of leukemic cells in the postremission phase to guide therapy, and expanded indications for allo-SCT (including older patients).^3,4^ However, the 5-year survival after diagnosis remains approximately 25%–30%,^5^ with 5-year mortality exceeding 50% among younger adults (<60 years old).^6^

Relapse is a major cause of morbidity and death among AML patients who have attained CR after the initial chemotherapy. The duration of relapse-free survival after relapse is typically short with 5-year mortality approaching 90%.^7^ Remission maintenance therapy, which aims to reduce the incidence of relapses in the postconsolidation phase, is thus a vital aspect of AML therapy, in particular for patients who are not candidates for immediate allo-SCT.

Avoiding relapse of AML has been more challenging than intuitively anticipated, and early trials using strategies that aimed at reducing relapse risk did not significantly alter routine practice.^8^ However, renewed interest in relapse prevention is demonstrated by recent large trials that have evaluated the fms-related tyrosine kinase 3 (FLT3) inhibitors midostaurin^9^ and quizartinib^10^ as well as an oral formulation of azacitidine^11^ in the postconsolidation phase. The RATIFY (midostaurin) and QuANTUM-First (quizartinib) phase III trials comprised treatment with FLT3 inhibitors during initial chemotherapy and in the post-CR phase, whereas the QUAZAR phase III trial assessed the efficacy of oral azacitidine only in the postremission phase. For an account of additional trials on remission maintenance strategies, readers are referred to de Lima et al.^12^

A phase III trial (the 0201 trial) evaluated the safety and relapse-preventive efficacy of combinatorial immunotherapy with histamine dihydrochloride (HDC) and low-dose interleukin-2 (IL-2) in AML patients after the completion of consolidation chemotherapy.^13^ The trial recruited 320 adult patients in CR regardless of previous induction or consolidation therapy (excluding previous allo-SCT) and met the primary endpoint of improved leukemia-free survival (LFS, defined as relapse or death, whichever occurred first) at >36 months. In 2008, HDC in conjunction with low-dose IL-2 was approved as remission maintenance therapy for AML patients in first CR (CR1) by the European Medicines Agency (EMA).^14^ Ensuing translational studies implied that HDC/IL-2 targets immunosuppressive myeloid cells, including myeloid-derived suppressor cells (MDSC), in the leukemic microenvironment (HDC component) with simultaneous activation of anti-leukemic functions exerted by natural killer (NK) cells and cytotoxic T lymphocytes (IL-2 component), enabling boosted antileukemic activity and protection against relapse.^15–17^ Recently, further analyses of the clinical benefit of HDC/IL-2 for relapse prevention have aimed at identifying responding subgroups of patients.^16–20^ Here, we review these studies alongside other currently approved relapse-preventive strategies in AML.

## FLT3 inhibitors and hypomethylating agents for AML remission maintenance

### Does targeted therapy benefit patients with *FLT3*-mutated AML in the postchemotherapy phase?

The type 1 FLT3 inhibitor midostaurin is approved by the EMA and the US Food and Drug Administration (FDA) for upfront therapy as well as remission maintenance in patients with *FLT3*-mutated AML. The pivotal RATIFY phase III trial evaluated overall, event- and disease-free survival among adult patients (*n* = 717, <60 years old) who received oral midostaurin or placebo in addition to standard chemotherapy^9^ (reviewed in Zhao et al.^21^). In the first phase of treatment, midostaurin was added at the end of each cycle of induction and consolidation chemotherapy. In a second phase, 205 patients who remained in CR after the completion of consolidation continued midostaurin or placebo for assessment of relapse and death.^22^

The trial demonstrated significantly superior overall survival (OS, primary endpoint, hazard ratio (HR) 0.78, *p* = 0.009) and disease-free survival for patients in the midostaurin arm.^9^ Anemia and rash were more common in the treatment arm, but the trial report did not specify occurrence of these toxicities in the postremission phase. In the RATIFY trial, no second randomization was performed for patients who had attained CR and there remains uncertainty whether midostaurin was beneficial in the remission maintenance phase or whether the improved outcome resulted mostly or solely from the inclusion of midostaurin during initial cycles of chemotherapy.^22–24^ The ambiguity regarding the value of midostaurin in the postconsolidation phase is reflected in current treatment recommendations: European LeukemiaNet guidelines recommend continued treatment also after the completion of consolidation,^2^ whereas midostaurin for remission maintenance is not listed in the guidelines of the American Society of Hematology for AML management.^25^

Building on the concept of FLT3 inhibition in *FLT3*-mutated AML, the potent and selective FLT3 type 2 inhibitor quizartinib was recently evaluated for efficacy in terms of OS prolongation in patients with *FLT3*-ITD^+^ AML (*n* = 539, 18–75 years old). The QuANTUM-First phase III trial largely recapitulated the design of the RATIFY trial using oral quizartinib or placebo at the end of the initial courses of chemotherapy followed by continued quizartinib monotherapy or placebo in the postconsolidation phase for patients who had achieved CR.^10^ In overall analysis, oral quizartinib was superior to placebo in terms of OS (primary trial endpoint, HR 0.78, *p* = 0.03), and an exploratory analysis of relapse-free survival (RFS) in patients who had achieved CR during induction showed a strong trend toward improved outcomes in the treatment arm (*n* = 297; RFS, HR 0.61), although there was no second randomization in the post-CR phase. Neutropenia (20%) was more common in the treatment arm and managed by dose reductions or treatment interruption. Quizartinib was also associated with QT prolongation (14%, mostly asymptomatic or grades 1–2). In contrast to the RATIFY trial, the QuANTUM-First trial also included patients >60 years old, constituting 40% of the study population; however, a posthoc analysis did not demonstrate significant overall benefit of treatment in the older patient group (*n* = 216; OS, HR 0.91). These results formed the basis for the recent approval of quizartinib for use with standard initial chemotherapy and as maintenance monotherapy after the completion of consolidation chemotherapy by the EMA and the FDA.

### Oral azacitidine (CC-486) for remission maintenance in older AML patients

An oral formulation of azacitidine, a hypomethylating epigenetic modifier that reduces silencing of tumor suppressor genes in leukemic cells, is approved by the EMA and the FDA for remission maintenance in AML. In the QUAZAR phase III trial, 472 patients (55–86 years old) in first CR were randomly assigned to receive oral azacitidine (CC-486) or placebo as maintenance therapy following the completion of initial chemotherapy.^11^ Enrollment included patients with intermediate or unfavorable risk with <4 months allowed between the achievement of CR and onset of oral azacitidine. All patients underwent induction chemotherapy; 65% received 0 or 1 courses of consolidation chemotherapy, and oral azacitidine was administered to assigned patients once daily for 14 days in 28-day cycles in the postconsolidation phase.

In overall analysis, patients in the treatment arm showed significantly longer median OS (25 months vs 15 months, *p* < 0.001, log-rank test) and RFS (10 months vs 5 months, *p* < 0.001) than patients in the placebo arm.^11^ Neutropenia (grades 3 or 4) occurred in 41% of treated patients versus 24% of controls. Nausea (65%), vomiting (60%), and diarrhea (50%) were common in treated patients, but the gastrointestinal side-effects typically subsided after initial cycles of treatment, and grade 3 or 4 gastrointestinal events were less common (5% in the treatment arm and <1% in controls). Serious infections were reported in 17% of treated patients and in 8% of controls.

Subsequent analyses of trial results addressed treatment efficacy by mutational status of leukemic cells at diagnosis. In patients with *NPM1*^+^ AML (*n* = 137, including patients with concomitantly mutated *FLT3*), median OS and RFS were significantly prolonged in the treatment arm (47 months vs 16 months, *p* = 0.038 for OS; 23 months vs 7 months, *p* = 0.005 for RFS) albeit with trends toward converging outcomes at late time points.^26^ The prolongation of median OS was most pronounced among *NPM1*^+^ patients positive for measurable residual disease (MRD) after initial chemotherapy (induction ± consolidation). The benefit of oral azacitidine for relapse prevention in older patients is further bolstered by a phase III study (HOVON97) assessing postremission parenteral administration of azacitidine that also showed significant reduction of relapse risk in patients ⩾60 years old albeit with a weaker trend for OS.^27^

## Immunotherapy for AML remission maintenance

### Previously evaluated immunotherapies for relapse prevention

The contribution by grafted T and NK cells to the anti-leukemic efficiency of allo-SCT has inspired the development of strategies aiming to reproduce the graft-versus-leukemia effect also in nontransplanted patients. As reviewed elsewhere,^8,12,15^ immunotherapy using single-agent interferon-α (IFN-α; an endogenous activator of NK cells),^8^ multiple regimens of monotherapy with IL-2 (an activator of NK and T cell functions),^28^ hybrid chemo-immunotherapy with anti-CD33 conjugated to a chemotherapeutic agent^29^ or linomide (a synthetic NK cell activator),^30^ did not reduce relapse risk in phase III trials when administered to AML patients in the postconsolidation phase. Additionally, nivolumab, which targets the PD-1/PD-L1 axis to revert inactivation of T cells, was reported not to reduce relapse risk in a randomized phase II trial.^31^ Recent reviews address the topic of new immunotherapeutic strategies in AML,^32–37^ but thus far only HDC/IL-2 is approved for remission maintenance.

### Targeting myeloid cell-induced immunosuppression for improved immunotherapy

The lack of clinically relevant benefit of previously evaluated pharmacological immunotherapies for relapse prevention may be unexpected not only because patients in CR harbor a low or even undetectable burden of leukemia—which intuitively would be accessible for elimination by activated immunity—but also because primary human AML cells often are sensitive to killing by autologous T and NK cells.^15,38^ These findings point to mechanisms of immunosuppression that compromise T and NK cell-mediated clearance of leukemic cells in patients, thus abating the efficiency of immunotherapy.

The NOX2 enzyme is expressed by myeloid cells, including MDSC and monocytic AML cells,^39,40^ and generates immunosuppressive reactive oxygen species (ROS); these oxygen derivatives are pivotal in the anti-microbial defense system but efficiently deactivate adjacent NK cells and T cells.^41^ The histamine H_2_ receptor (H_2_R) is co-expressed with NOX2 on myeloid cells, and agonist activity at H_2_R markedly reduces NOX2-derived ROS formation.^39,42^ By agonistic binding to H_2_Rs, HDC has been shown to rescue NK cells and T cells from deactivation in a NOX2^+^ microenvironment and thus restore the immune activating capacity of IL-2.^43,44^ The NOX2-inhibitory action of HDC or other H_2_R-selective agonists thus reportedly synergizes with IL-2 to eliminate neoplastic cells, including myeloid leukemic cells, in vitro^45,46^ and in murine models in vivo.^47,48^

### Phase III trial of HDC and low-dose IL-2 for AML remission maintenance

Combinatorial remission maintenance immunotherapy with HDC and low-dose IL-2 was evaluated in a phase III trial of 320 adult AML patients in 11 countries (the 0201 trial).^13^ Enrolled patients (*n* = 320, 18–84 years, median 57 years) were in confirmed CR1 (*n* = 261) or any subsequent CR (CR > 1, *n* = 59) and not eligible for upfront allo-SCT. Patients were randomly assigned to receive HDC/IL-2, with no treatment (standard of care or observation) as the comparator. In the treatment arm, patients received ten 3-week cycles of HDC/IL-2 (0.5 mg HDC, 16 400 U/kg IL-2) s.c. twice daily with 3–6 weeks of rest between cycles for 18 months; patients who completed the regimen were thus treated for a total of 30 out of 78 weeks. The low dose of IL-2 given (approximately 1MIU bid) was deemed suitable for unsupervised home treatment based on previous phase I/II studies.^49^

The primary analysis was performed when all patients had been followed for hematological relapse or death for at least 36 months after random assignment (median follow-up 48 months). The primary endpoint was LFS, defined as time from random assignment to relapse or death from any cause, among all patients. Secondary endpoints included OS among all patients, efficacy in patients in CR1 or CR > 1, and efficacy in patients who were above or below 60 years old at enrollment.

The trial reached the primary endpoint of improved LFS at 36 months among all patients (HR 0.71, *p* = 0.008), which remained significant after correction for predefined confounders (HR 0.69, corrected *p*-value 0.006; Table 1). The treatment arm was significantly superior for LFS in three independent participating countries, arguing against unknown imbalances between study arms.^50^ LFS was additionally improved by HDC/IL-2 for the secondary endpoints of efficacy in patients in CR1 (HR 0.69, *p* = 0.01) and in those <60 years old (CR1; HR 0.56, *p* = 0.004; Table 1), but the treatment did not significantly reduce relapse risk in older patients (>60 years old). The trial did not reach the secondary endpoint of OS for all randomized patients (Table 1); however, effects of HDC/IL-2 on HRs for LFS and OS were correlated (*R*^2^ = 0.93 for all patients, *R*^2^ = 0.88 for patients in CR1).^51^

**Table 1.** Primary, secondary, and post hoc analyses of HDC/IL-2 efficacy for AML remission maintenance in the 0201 trial.

AML patient group	LFS^a^				OS^a^			
	Log-rank, *p*	HR	95% CI^b^	3 Year survival rate, %^c^	Log-rank, *p*	HR	95% CI^b^	3 Year survival rate, %^c^
All patients	**0.01** ^d^	**0.71**	**0.54–0.92**		*0.21* ^d^	*0.82*	*0.61–1.11*	
Control, *n* = 160				**24.1**				*44.1*
HDC/IL-2, *n* = 160				**34.1**				*47.6*
CR1	*0.01* ^e^	*0.69*	*0.51–0.93*		*0.16* ^e^	*0.78*	*0.56–1.09*	
Control, *n* = 132				*26.0*				*46.3*
HDC/IL-2, *n* = 129				*40.1*				*54.7*
Chemosensitive AML^f^	0.01	0.65	0.46–0.92		0.09	0.71	0.48–1.05	
Control, *n* = 99				29.7				49.7
HDC/IL-2, *n* = 104				45.1				59.3
Normal karyotype AML	0.02	0.58	0.37–0.91		0.13	0.66	0.39–1.13	
Control, *n* = 67				27.7				52.6
HDC/IL-2, *n* = 61				50.0				63.0
FAB M4/M5^g^	0.07	0.62	0.37–1.04		0.32	0.74	0.41–1.35	
Control, *n* = 44				29.5				47.7
HDC/IL-2, *n* = 46				47.8				62.7
CR1 <60 years	*0.004*	*0.56*	*0.38–0.84*		*0.07*	*0.65*	*0.41–1.05*	
Control, *n* = 81				*28.9*				*52.7*
HDC/IL-2, *n* = 79				*50.5*				*65.1*
Chemosensitive AML^f^	0.001	0.48	0.30–0.76		0.02	0.53	0.31–0.92	
Control, *n* = 64				32.0				54.2
HDC/IL-2, *n* = 66				55.9				70.4
Normal karyotype AML	0.006	0.40	0.20–0.79		0.04	0.43	0.18–1.01	
Control, *n* = 37				31.3				58.7
HDC/IL-2, *n* = 35				65.6				76.5
FAB M4/M5^g^	0.006	0.39	0.20–0.78		0.05	0.44	0.19–1.03	
Control, *n* = 26				26.9				50.0
HDC/IL-2, *n* = 32				62.5				77.6

Bold figures refer to the primary trial endpoint^13^ and italic figures to secondary endpoints^13,52^; remaining figures represent post hoc analyses.^18,19,40^

a LFS, defined as time from random assignment to relapse or death whichever comes first; OS from random assignment.

b Confidence intervals for the hazard ratios.

c Kaplan–Meier estimates at 3 years from random assignment in each treatment arm.

d Stratified by country and CR stratum (CR1 or CR > 1).

e Stratified by country.

f Defined by achievement of CR after the first cycle of induction chemotherapy.

g Microscopical appearance of leukemic cells by FAB classification. M4 = acute myelomonocytic leukemia, M5 = acute monocytic leukemia.

AML, acute myeloid leukemia; CR, complete remission; FAB, French-American-British; HDC/IL-2, histamine dihydrochloride and low-dose interleukin-2; LFS, leukemia-free survival; OS, overall survival.

Among phase III trial patients who were randomly assigned to HDC/IL-2 or control after having attained CR after one or several previous relapses (CR > 1, referring to patients in CR2 or any subsequent CR, *n* = 59), a predefined secondary endpoint of prolonged duration of CR > 1 versus the preceding CR (“remission inversion”) significantly favored the treatment arm (*p* = 0.03).^52^

The trial reported no treatment-related mortality and a similar frequency of grades 3 and 4 events in both study arms.^13^ Patients in the HDC/IL-2 arm experienced transient flush, mild hypotension, transient headache, eosinophilia (albeit devoid of related morbidity), transient low-grade fever, and transient injection site reactions to IL-2. Quality-of-life (QoL) during treatment was self-assessed by patients using the EORTC QLQ-C30 (v2) instrument (global health, fatigue, pain, nausea), and a comparative analysis did not indicate QoL differences between study arms.^53^ Ninety-two percent of patients who remained in CR completed treatment as prescribed (10 cycles), although 26% required temporary dose reductions of study medications, especially during early treatment cycles.^13^

To address the question whether the benefit of HDC/IL-2 might have been mediated solely by the IL-2 component, Berry et al. employed two Bayesian hierarchical models to analyze relapse prevention by HDC/IL-2 versus IL-2 in meta-analysis of data from six randomized trials (*n* > 1500).^54^ The results showed that HDC/IL-2 significantly improved LFS over standard of care in both models, whereas monotherapy with IL-2 did not. In addition, the 5-year cumulative probability of HDC/IL-2 being superior to IL-2 alone in Model 2 was 0.956, supporting the contribution by the HDC component for the efficacy of this combinatorial treatment strategy. HDC/IL-2 was approved as AML remission maintenance therapy in the European Union in 2008 with a confirmatory approval granted in 2018.^14^

### Aspects of anti-leukemic immunity determine the clinical efficacy of HDC/IL-2

As a postapproval requirement by the EMA, the Re:Mission single-arm phase IV trial, performed in four European countries, assessed the immunomodulatory effects of HDC/IL-2.^16,17,55–60^ In this trial, AML patients (*n* = 84, age 18–79, all in CR1) received ten 3-week cycles of HDC/IL-2 (the same schedule and dosing as in the phase III trial) with peripheral blood mononuclear cells collected before and after treatment cycles 1 and 3.

**Figure 1.**
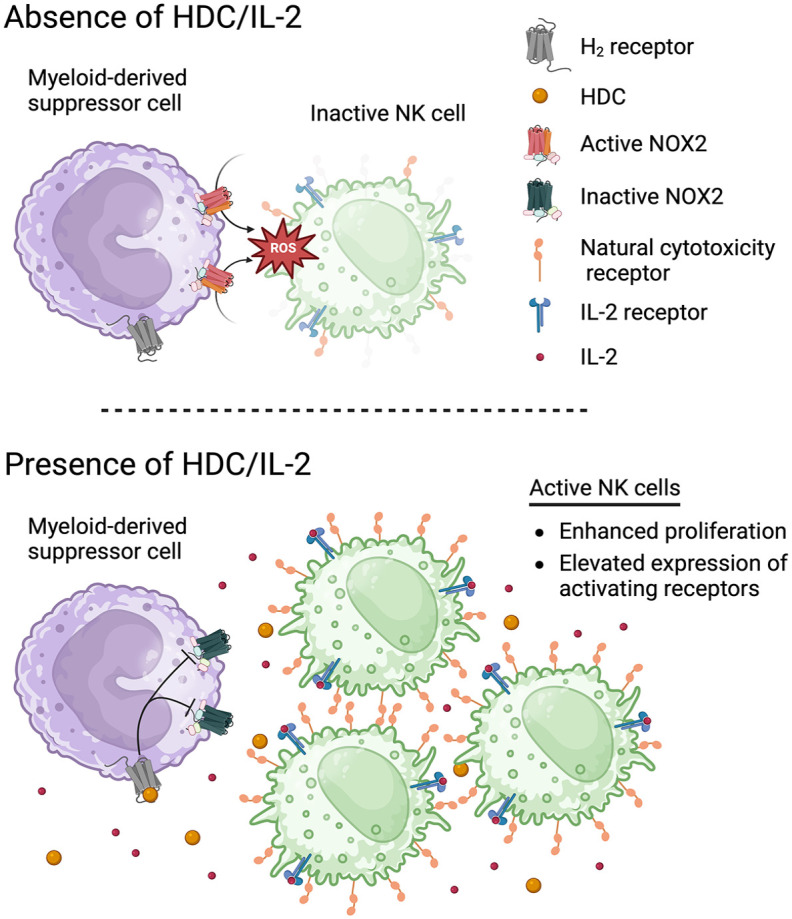
Proposed mode of action of HDC/IL-2 in acute myeloid leukemia. HDC suppresses formation of immunosuppressive NOX2-derived ROS produced by myeloid cells, including MDSC and monocytic AML cells. HDC acts by agonist activity at H_2_R that are co-expressed with NOX2 on the myeloid cells. The achieved protection of NK cells translates into improved efficacy of immunomodulatory strategies, including low-dose IL-2, enabling anti-leukemic NK cell activity and protection against relapse. Source: Created in BioRender. Nilsson, M. (2025) https://BioRender.com/ecfwr5t AML, acute myeloid leukemia; H_2_R, histamine H_2_ receptors; HDC/IL-2, histamine dihydrochloride and low-dose interleukin-2; MDSC, myeloid-derived suppressor cells; NK, natural killer; ROS, reactive oxygen species.

The trial results implicated NK cell function in the clinical efficacy of HDC/IL-2. During cycles of HDC/IL-2 peripheral blood NK cell counts increased 2.5-fold with parallel induction of surface expression of natural cytotoxicity receptors (NCR; NKp30 and NKp46). In addition, induction of NK cell NCR expression during initial treatment cycles coincided with reduced risk of relapse.^16,55^ More recent analyses of the Re:Mission trial data investigated the impact of refined aspects of NK cell biology and showed that genetic determinants leading to an NK cell repertoire of immature, cytokine-responsive NK cells were associated with favorable clinical outcome.^61–64^ Figure 1 depicts the proposed antileukemic mechanism of HDC/IL-2 in the AML remission maintenance setting.

**Figure 2.**
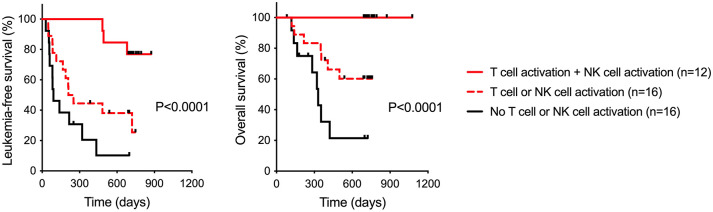
Immunomodulation versus outcome of HDC/IL-2 treatment in a phase IV trial of relapse prevention in AML. Data show leukemia-free and overall survival in HDC/IL-2-treated patients achieving NK cell activation or T cell activation (high expression of the NCR NKp46 or transition from effector memory to cytotoxic effector T cells; *n* = 16), both (*n* = 12) or neither (*n* = 16) as indicated. Leukemia-free survival is defined as time from random assignment to relapse of AML or death from any cause. Statistics by the logrank test for trend. Source: Adapted from Sander et al.^17^ AML, acute myeloid leukemia; HDC/IL-2, histamine dihydrochloride and low-dose interleukin-2; NCR, natural cytotoxicity receptor; NK, natural killer.

The Re:Mission trial additionally addressed the dynamics of T cell phenotypes during therapy.^17^ During the first treatment cycle, approximately 40% of evaluable patients showed accumulation of CD8^+^ cytotoxic effector T cells (T_EFF_) in blood with parallel reduction of effector memory T cells (T_EM_). The treatment-induced transition of T_EM_ to T_EFF_ cells heralded strikingly favorable clinical outcome, including in older patients. A consorted analysis in all participating patients implied not only that treatment effects on NK cells and T cells each correlated positively with outcome but also that patients achieving NK cell activation in conjunction with T cell transition showed superior LFS and OS (Figure 2, adapted from Sander et al.^17^).

In addition to the effects on lymphocyte populations, blood counts of monocytic myeloid-derived suppressor cells (M-MDSC), an immature myeloid cell type that exerts immunosuppression partly by secreting ROS, were reduced by approximately 60%–70% during cycles of HDC/IL-2.^59^ Reduced counts of M-MDSC during early cycles of HDC/IL-2 significantly heralded prolonged LFS, providing additional support to the proposed mode of action of the treatment.

### Post hoc analyses of HDC/IL-2 efficacy by chemoresponsiveness and leukemic cell karyotype

In recent years, posthoc analyses of the HDC/IL-2 phase III trial data have served to further define responding subgroups of patients. These analyses involved assessment of efficacy according to leukemic cell chemosensitivity and karyotype.

Patients who require >1 course of induction chemotherapy to attain CR are at elevated risk for relapse and death.^65^ Among younger patients (<60 years old) in the phase III trial, failure to achieve CR1 after one induction course, likely reflecting reduced chemosensitivity of the leukemic cells, was observed in 22% of patients; in this subgroup HDC/IL-2 did not improve LFS or OS. By contrast, LFS and OS were significantly superior in the treatment arm among younger patients who had achieved CR1 after one course of induction (HR 0.48, *p* = 0.001 for LFS; HR 0.53, *p* = 0.02 for OS; Table 1).^19^

Normal karyotype AML, where leukemic cells carry morphologically and numerically intact chromosomes, comprises 40%–60% of adult AML. The most prevalent mutations, *NPM1* and *FLT3-*ITD, are present in 40%–50% and 30%–40% of AML cases in this patient group, respectively.^66,67^ Mutational characteristics of leukemic cells were unavailable in the 0201 phase III trial; however, posthoc analyses showed pronounced efficacy among younger patients (<60 years) with AML of normal karyotype.^18,68^ In these patients, treatment entailed significantly improved LFS (HR 0.40, *p* = 0.006, HDC/IL-2 vs control) and OS (HR 0.43, *p* = 0.04; Table 1). HDC/IL-2 did not improve outcomes in AML of any aberrant karyotype or among older patients with normal karyotype AML.^18^

The observed effect size of HDC/IL-2 over control in younger patients with normal karyotype AML (66% vs 31% LFS at 3 years; Table 1) implies that the treatment effect likely resides within at least one of the larger groups of patients with normal karyotype (*FLT3* and/or *NPM1*-mutated) AML. In the single-arm phase IV Re:Mission trial, 4/4 patients with *FLT3-*ITD^+^ AML relapsed within 200 days,^18^ suggesting that the anti-leukemic efficacy of HDC/IL-2 among normal karyotype patients instead may derive from *NPM1*-mutated AML. The view that HDC/IL-2 is efficacious in *NPM1*^+^ AML is bolstered by pronounced treatment efficacy in myelomonocytic/monocytic forms of AML (FAB classes M4/M5) where *NPM1* mutations accumulate^69^ (Table 1).

In the 0201 phase III trial, patients receiving HDC/IL-2 with normal karyotype or chemosensitive AML only rarely relapsed >12 months after random assignment suggesting durable protection against relapse.^18,19^ The exploratory nature of these posthoc analyses should be emphasized, and further studies are required to define the efficacy of HDC/IL-2 in molecular and other subgroups of AML.

### Assessment of molecular relapse during treatment with HDC/IL-2

In recent years, molecular MRD monitoring over the course of AML treatment has become common practice for informing further treatment decisions. Recently, a German/Austrian registry of patients <60 years old with *NPM1*-mutated AML in CR1 reported results of serial monitoring of MRD during treatment with HDC/IL-2. Using this registry, the 3306 study assessed the time from the completion of previous chemotherapy to switch from MRD negativity to positivity (“molecular relapse”) in HDC/IL-2-treated patients for comparison against carefully matched contemporary controls retrieved from the German-Austrian Cooperative Group registries.^20^ The results suggested that treatment with HDC/IL-2 reduced the incidence of molecular relapse in patients carrying *NPM1* mutations (*n* = 25, HR 0.28, *p* = 0.07 vs controls) and within the group of patients with myelomonocytic/monocytic *NPM1*-mutated AML (*n* = 15, HR 0, *p* = 0.03). HDC/IL-2 prevented late rather than early molecular relapses, with no molecular relapses (0/8 patients) occurring beyond 6 months in *NPM1-*mutated AML among HDC/IL-2-treated patients versus 9/14 (64%) in the matched controls (*n* = 22, HR 0, *p* = 0.009). There were no LFS events in the HDC/IL-2 group versus 4 LFS events, including two deaths, among matched controls. Although achieved within a small cohort, these results align with the proposed clinical efficacy of HDC/IL-2 in younger AML patients carrying *NPM1* mutations, including those with AML of myelomonocytic/monocytic morphology (FAB-M4/M5 AML).^42^

## An emerging landscape of relapse prevention in AML

For many decades, no further treatment with consensual clinical benefit was available for AML patients—not eligible for immediate allo-SCT and excluding acute promyelocytic AML—who had attained CR after the early phases of induction and consolidation chemotherapy, and several trials evaluating relapse-preventive strategies yielded disappointment. In recent years, FLT3 inhibitors, oral azacitidine and HDC/IL-2 have emerged as treatment options to avoid relapse in the postconsolidation phase. While the efficacy of midostaurin and quizartinib for remission maintenance in *FLT3*-mutated AML remains to be formally validated, oral azacitidine and HDC/IL-2 were only administered after the completion of chemotherapy in the pivotal phase III trials (QUAZAR and 0201, respectively) ascertaining that these regimens prevent relapse in the postchemotherapy phase.

Patient age may determine the relative benefit of oral azacitidine versus HDC/IL-2. While HDC/IL-2 was preferentially efficacious in patients <60 years old,^13^ oral azacitidine for remission maintenance was evaluated in older patients (>55 years old). A retrospective phase II trial using a similar remission maintenance strategy in younger patients—using cycles of parenteral decitabine, a structurally and pharmacodynamically related analog of azacitidine, for patients <60 years old versus historical controls—did not imply efficacy in terms of LFS or OS^70^ (further discussed in Patel et al.^71^). These findings highlight that the benefit of hypomethylating agents for relapse prevention in younger AML patients awaits confirmation.

Further studies are required to determine the respective efficacy of oral azacitidine and HDC/IL-2 within molecular subgroups of AML and in patients with variable intensity and efficiency of previous treatment. Notably, while azacitidine benefited patients who had achieved minimal prior chemotherapy and had pronounced efficacy among MRD positive patients, HDC/IL-2 was most efficacious in patients with a chemosensitive leukemic clone (likely reflecting reduced disease burden at the end of chemotherapy). Thus, further improvements to frontline AML therapy may allow HDC/IL-2 efficacy in additional AML subtypes. Also, the prospect of combining these or other treatments (including FLT3 inhibitors) may be contemplated. In this process, the evaluation of candidate relapse-preventive therapies may benefit from serial analysis of MRD (maintenance of MRD negativity and/or reversal of MRD positivity) during therapy and should consider patient characteristics including age, response to previous chemotherapy and morphological or molecular forms of leukemia. The clinical benefit of recent strategies for remission maintenance may thus inspire additional studies to complete an emerging landscape aiming to prevent relapse in larger groups of AML patients.

## Conclusion

Except for allo-SCT, relatively few treatment options are currently available to maintain durable CR in AML patients who have completed the initial phases of chemotherapy. While several novel immunotherapies have been developed, few have been evaluated for relapse-preventive efficacy in AML in randomized settings. Broad activators of NK cell (single-agent IL-2, IFN-α) and T cell (checkpoint inhibitors) function have hitherto shown little or no benefit in the postremission phase, and new immunotherapies will likely have to counter aspects of immunosuppression in the leukemic bone marrow. HDC/IL-2 is approved for maintenance of first CR in patients within the EU and was recently shown to reduce relapse risk in younger adults (<60 years old) with favorable outcome of previous chemotherapy and in AML of normal karyotype. The relative benefit of the currently approved maintenance therapies FLT3 inhibitors, oral azacitidine and HDC/IL-2 is likely dependent on disease characteristics including type of AML, intensity or effectiveness of previous therapy and age. Future studies of these and arising maintenance strategies—alone or in combination—might bring more efficient relapse prevention to a wider group of AML patients.

## Declarations

**Ethics approval and consent to participate:** Not applicable.

**Consent for publication:** Not applicable.

**Availability of data and materials:** Not applicable.
